# Draft Genome Sequence of Bacillus paranthracis Strain DB-4, Isolated from Nukadoko, Fermented Rice Bran for Japanese Pickles

**DOI:** 10.1128/MRA.00705-21

**Published:** 2021-10-07

**Authors:** Daisuke Fukuda, Cirilo Nolasco-Hipolito

**Affiliations:** a Vaccine and Infectious Disease Area, Medical Affairs, Merck Research Laboratories, MSD K.K., Chiyoda-ku, Tokyo, Japan; b Institute of Biotechnology, Universidad del Papaloapan Campus Tuxtepec, Tuxtepec, Oaxaca, México; University of Rochester School of Medicine and Dentistry

## Abstract

Bacillus paranthracis strain DB-4 was isolated from nukadoko in Japan. We report the draft genome sequence of this strain to provide insights into the survival mechanisms of lactic acid bacteria in fermented rice bran.

## ANNOUNCEMENT

The Bacillus cereus group, also known as B. cereus sensu lato, comprises at least 21 related species (1). Members of the B. cereus group, including Bacillus paranthracis, are Gram-positive, facultatively anaerobic, and spore-forming. Due to their variety of lifestyles and genetic diversity, some strains of the B. cereus group are considered to be beneficial for human and animal health (2, 3). *Bacillus* spp. may also protect lactic acid bacteria in fermented foods from reactive oxygen species through their catalase activity (4, 5). To clarify the physiological role of *Bacillus* spp. in fermented foods, we analyzed the genome of *B. paranthracis* DB-4, isolated from homemade nukadoko, fermented rice bran used as a bed for making Japanese pickles.

*B. paranthracis* DB-4 was first discovered in homemade nukadoko in Yokohama, Japan. Strain DB-4 was isolated by spreading serially diluted nukadoko onto MRS agar (Difco) at 30°C for 2 days. A single colony of strain DB-4 was picked for culturing prior to DNA isolation.

Strain DB-4 was cultured in MRS broth (Difco) at 30°C for 15 h (late log phase). Then, genomic DNA was extracted from *B. paranthracis* DB-4 and purified using the GeneJET genomic DNA purification kit (Thermo Fisher), following the instruction manual. After the quality and quantity of the genomic DNA obtained were checked using a Synergy LX microplate reader (BioTek, USA) and the QuantiFluor double-stranded DNA (dsDNA) system (Promega), respectively, the sequence library was prepared using a Nextera XT library preparation kit (Illumina). The quantity and quality of the library were verified using the Agilent fragment analyzer and dsDNA 915 reagent kit (Advanced Analytical Technologies). Whole-genome sequencing was performed using the Illumina MiSeq sequencer with 2 × 250-bp paired-end reads. The sequences were processed to remove low-quality bases using Cutadapt ver. 1.9.1 (6) and Sickle ver. 1.33 (https://github.com/najoshi/sickle). Sequencing resulted in 5,085,667 paired-end reads. *De novo* genome assembly was performed using the SPAdes ver. 3.13.2 genome assembler (5, 7) with default parameters.

The assembly yielded 57 contigs covering a total of 5,424,208 bp, with an *N*_50_ value of 498,305 bp and a G+C content of 35.2%. The genome sequence was annotated using the DFAST ver. 1.14.5 annotation pipeline (8, 9), which revealed 5,595 coding DNA sequences (CDSs), 103 tRNA genes, 13 rRNA genes, 1 CRISPR, and a coding ratio of 84.0%. The average nucleotide identity (ANI) was analyzed using the JSpeciesWS online service (10), which showed 98.9% and 91.96% identities with the genomes of *B. paranthracis* Mn5^T^ (GenBank accession number GCA_001883995.1) and B. cereus ATCC 14579^T^ (GCA_000007825.1), respectively. These ANI scores indicate that strain DB-4 belongs to the species *B. paranthracis*.

Five catalase genes, including vegetative catalase genes and a manganese catalase gene, were identified in the genome of *B. paranthracis* DB-4. A putative heme-dependent peroxidase gene and three superoxide dismutase genes were also identified in the genome of DB-4. These may contribute to eliminating reactive oxygen species in fermented nukadoko.

The whole-genome sequence, assembly, and annotation of *B. paranthracis* DB-4 will support our understanding of the physiological roles of B. cereus group bacteria in fermented foods, lactate fermentation processes, and human health (7, 11).

### Data availability.

The genome sequence was deposited in the DDBJ/ENA/GenBank under accession number BPLB00000000.1. The draft genome project data have been submitted under BioProject accession number PRJDB11683, DRA accession number DRR294728, and Sequence Read Archive (SRA) accession number DRX284184.
